# The Blending Effect of Single-Shade Composite with Different Shades of Conventional Resin Composites—An *In Vitro* Study

**DOI:** 10.1055/s-0042-1744369

**Published:** 2022-06-21

**Authors:** Md Sofiqul Islam, Noorul Huda, Sankavi Mahendran, Smriti Aryal AC, Mohannad Nassar, Mohammed Mustahsen Rahman

**Affiliations:** 1 Department of Operative Dentistry, RAK College of Dental Sciences, RAK Medical and Health Sciences University, Ras Al-Khaimah, United Arab Emirates; 2 Department of Clinical Dentistry, RAK College of Dental Sciences, RAK Medical and Health Sciences University, Ras Al-Khaimah, United Arab Emirates; 3 Department of Oral and Craniofacial Health Sciences, College of Dental Medicine, University of Sharjah, Sharjah, United Arab Emirates; 4 Department of Preventive and Restorative Dentistry, College of Dental Medicine, University of Sharjah, Sharjah, United Arab Emirates; 5 Department of Periodontology, RAK College of Dental Sciences, RAK Medical and Health Sciences University, Ras Al-Khaimah, United Arab Emirates

**Keywords:** OMNICHROMA, blending effect, color stability, resin composite, single-shade composite, Beautifil II Enamel

## Abstract

**Objectives** Single-shade composite systems are gaining popularity among clinicians due to the claimed potential of blending with different tooth structure shades while restoring the tooth. The purpose of this study was to evaluate the blending effect of two single-shade composite with different shades of conventional resin composite systems.

**Materials and Methods** Seventy-two composite cylinders of B1, B2, A1, A2, A3, or A3.5 shade from CharmFil Plus (CP) and Filtek Universal Restorative (3M) were prepared using custom-made silicone mold. Single-shade composite OMNICHROMA (OC) or Beautifil II Enamel (BE) was placed in the center of each cylinder and polymerized. The color parameters, lightness (L*), chroma (C*), and hue (H*) of each composite were measured using a color chronometer. Furthermore, color stability of the samples was evaluated after 1-week staining challenge.

**Statistical Analysis** Multivariant analysis was performed to evaluate the effect of material and shade on the color parameters. Multiple comparisons of the data were performed using post hoc test. The staining challenge data were analyzed using repeated measure analysis of variance and paired sample *T* -test.

**Results** The multivariant analysis showed a statistically significant difference in color parameters among CP, 3M, OC, and BE ( *p* = 0.001). Image analysis showed a visual blending effect for both OC and BE for certain shades; however, some color contrast with the darker shades was observed. The C* value of OC showed a similar pattern to CP; however, the H* of the latter was closely followed by BE. The L* value showed statistically significant difference among the shades of 3M, and in OC and BE when blended with 3M.

**Conclusion** All four materials used in this study showed color alteration after the staining challenge. Single-shade composite can blend with only certain shades of resin composites.

## Introduction

In order to satisfy the growing demands for esthetic restorations that have natural tooth-like appearance, the research and development of this field is expanding in an exponential rate. Several research attempts have been made within the scholarly literature in the past few decades to enhance the quality of bonding and for better adaptation between the tooth structure and resin-based restorative materials. 1 Over the years, resin composite systems have improved remarkably which led to the gradual replacement of amalgam with these systems. The current technology utilized to produce and incorporate the filler into the resin matrix enables attaining superior esthetics, better wear resistance properties, and optimal compressive strength for restoring anterior and posterior teeth. 2

Natural teeth express variable colors at different parts, and thus, it is crucial to match these colors before placing a new restoration or repairing an existing one. The achievement of masterful esthetic results with the use of resin composite filling materials is closely related to the color properties of these materials. 3 This leads the manufacturers and clinicians to work on developing techniques and materials that result in mimicking the natural dentition by optimizing the color properties of the used restorative materials. 4 5 The color-related properties of resin composite materials are ascribed as color compatibility which denotes to matching between the restorative material and the tooth structure, color stability in different stages such as during and after manufacturing or placement, and color interaction which is related to the layering and color shifting where the surrounding hard tissue emits similar color to the composite materials. 6 7 The blending effect, color induction, and color assimilation effects of resin composite are collectively known as the chameleon effect, 8 9 which enables resin-based restorative materials to obtain a shade that bears a resemblance to the color of the surrounding tooth structure. 10 In order to be considered as an esthetic restorative material, the resin composite needs to blend with its adjoining structures. Resin composite materials allow light scattering which produces internal diffusion to enamel and dentin, thus aiding the resin composite to blend with the surroundings. The color alteration of esthetic restorations hugely depends on light scattering and absorption coefficients to make it undetectable by blending with the tooth structure. 11

The observable color of a dental restoration is dictated by the light scattering properties of the resin-based restorative material and the substrates beneath the restoration. 12 Natural teeth and resin composite share three basic color parameters termed as lightness (L*), chroma (C*), and hue (H*). Other color parameters such as opacity, translucency, fluorescence, and surface gloss necessitate optimization during the manufacturing process of resin-based composite materials. 13 OMNICHROMA (OC) is one of the latest advancements in the field of dental materials that is designed to embody the color blending potential with different shades of natural tooth structure using a single-shade composite. 14 OC is the first omnichromatic resin-based composite material that is claimed to have an inherent color matching potential with various tooth or conventional composite shades. 15 Before the invention of single-shade composite systems, multiple shades of the conventional composite were required to reach an esthetically appealing final result of the restorative work. OC created a unique opportunity for clinicians to reduce the need to store multiple shades which would positively impact the materials' total cost and management at the dental office. 16

Beautifil II Enamel (BE) is a distinctive addition to dentin shades of nanohybrid composite that poses an optical characteristics of the enamel tones, thus allows creating polychromatic restorations with morphology and esthetics of natural teeth. 17 The potential of single-shade resin composite to blend with multiple shades and various teeth colors demonstrates a notable evolution in resin composite restoration technology which will eventually help clinicians obtain better esthetic results by minimizing the number of technical errors during shade selection.

The aim of the current research was to evaluate the blending effect of OC and BE with two brands of multishaded resin composite, namely, CharmFil Plus (CP) and Filtek Z250XT (3M). The color compatibility, stability, and interactions of OC and BE were evaluated by analyzing the color parameters after polymerization and after staining challenge while surrounded by various shades of conventional resin composite. The null hypothesis tested in this study were (1) OC and BE cannot blend with different shades of CP and 3M; (2) there are no differences in color blending capability between OM and BE; and (3) there are no effect of staining challenge on the color parameters of the tested resin composite systems.

## Materials and Methods

### Preparation of Resin Composite Cylinder

Seventy-two polymerized resin composite cylinders used in this study were prepared using CharmFil Plus by Dentkist Inc, Korea or Filtek Z250XT Universal Restorative by 3M, United States. Round-shaped composite cylinder (10 mm in diameter and 3 mm height) with 1 mm deep and 5 mm circular-shaped empty space was created for the test material using custom-made silicon mold. Six shades, B1, B2, A1, A2, A3, or A3.5, of each composite were used. The color parameters, L*, C*, and H*, of the base composite were measured using a digital color chromometer VITA Easyshade Advance 4.0 (VITA Zahnfabrik, Germany). For accuracy, each specimen was measured in triplicate.

### Blending Effect

The open spaces of base composite cylinder from each shade ( *n* = 6) were filled with either OC single-shade universal composite by Tokuyama Dental, Japan or with BE translucent shade Y2250 by SHOFU INC, Japan. The color parameters, L*, C*, and H*, of the test materials were measured after light curing as per the manufacturers' instructions. For accuracy, each specimen was measured in triplicate. The blending of test composite with base composite was determined by comparing the color parameters of test composite with that of base composite for each shade.

### Staining Challenge

After measuring the blending capability, the composite cylinders were used in the staining challenge. For the staining challenge, 100 mL of boiling water was taken in a glass beaker with 2 g of dry black tea leaves and kept for 10 minutes after which the tea leaves were removed. Each specimen was immersed individually in 3 mL of staining solution for 1 week continuously without changing the solution. The values were remeasured for each specimen in triplicate.

### Statistical Analysis

The raw data were analyzed using statistical software (SPSS 16.0). The multivariant analysis was performed to evaluate the effect of materials and shade on the color parameters. Multiple comparisons of the data were performed using post hoc test. The staining challenge data were analyzed using repeated measure analysis of variance (ANOVA) and paired sample *T* test.

## Results

### Blending Effect with CP

**Fig. 1:**
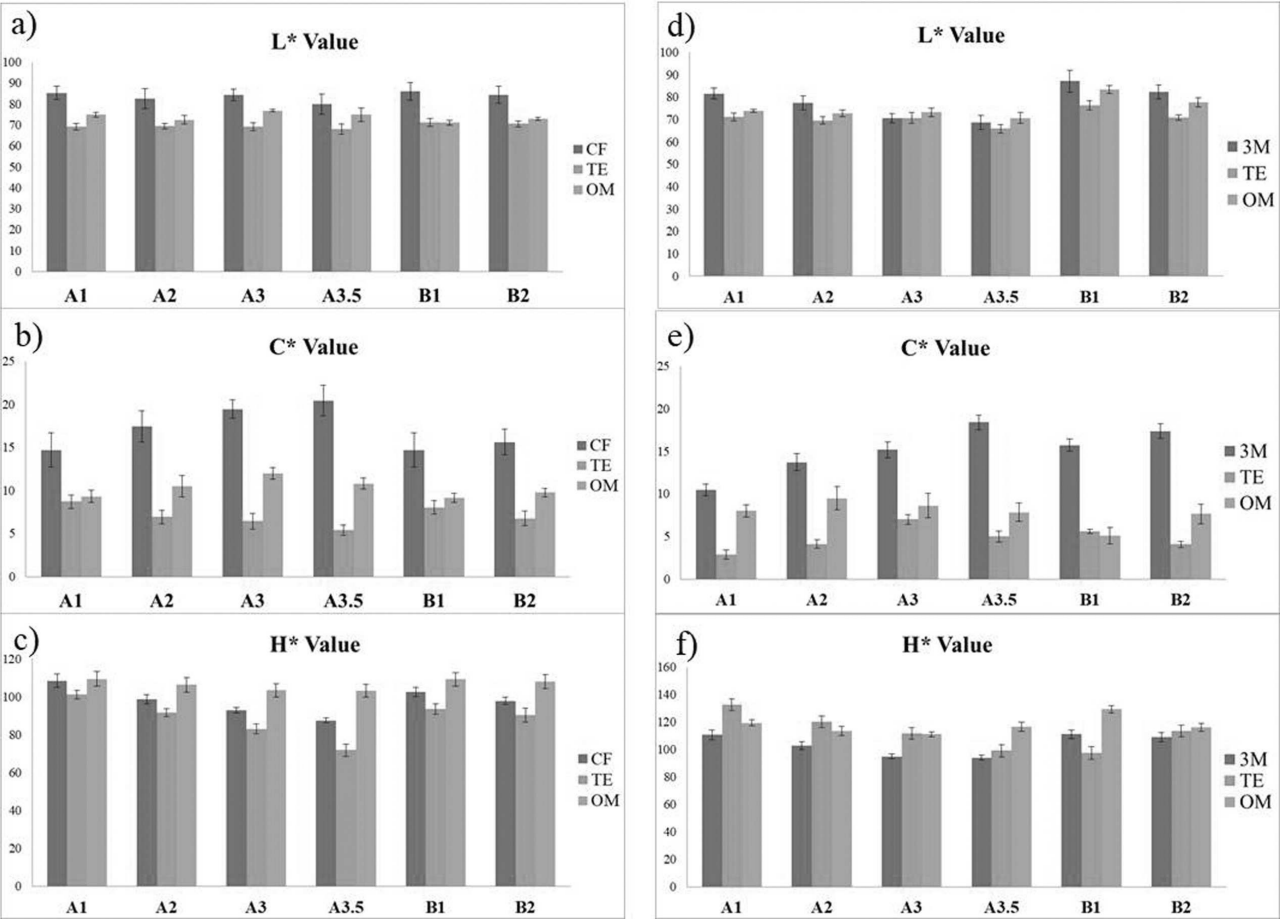
(A–C) The lightness, chroma, and hue of CF with BE and OM. (D–F) The lightness, chroma, and hue of 3M with BE and OM.

**Fig. 2:**
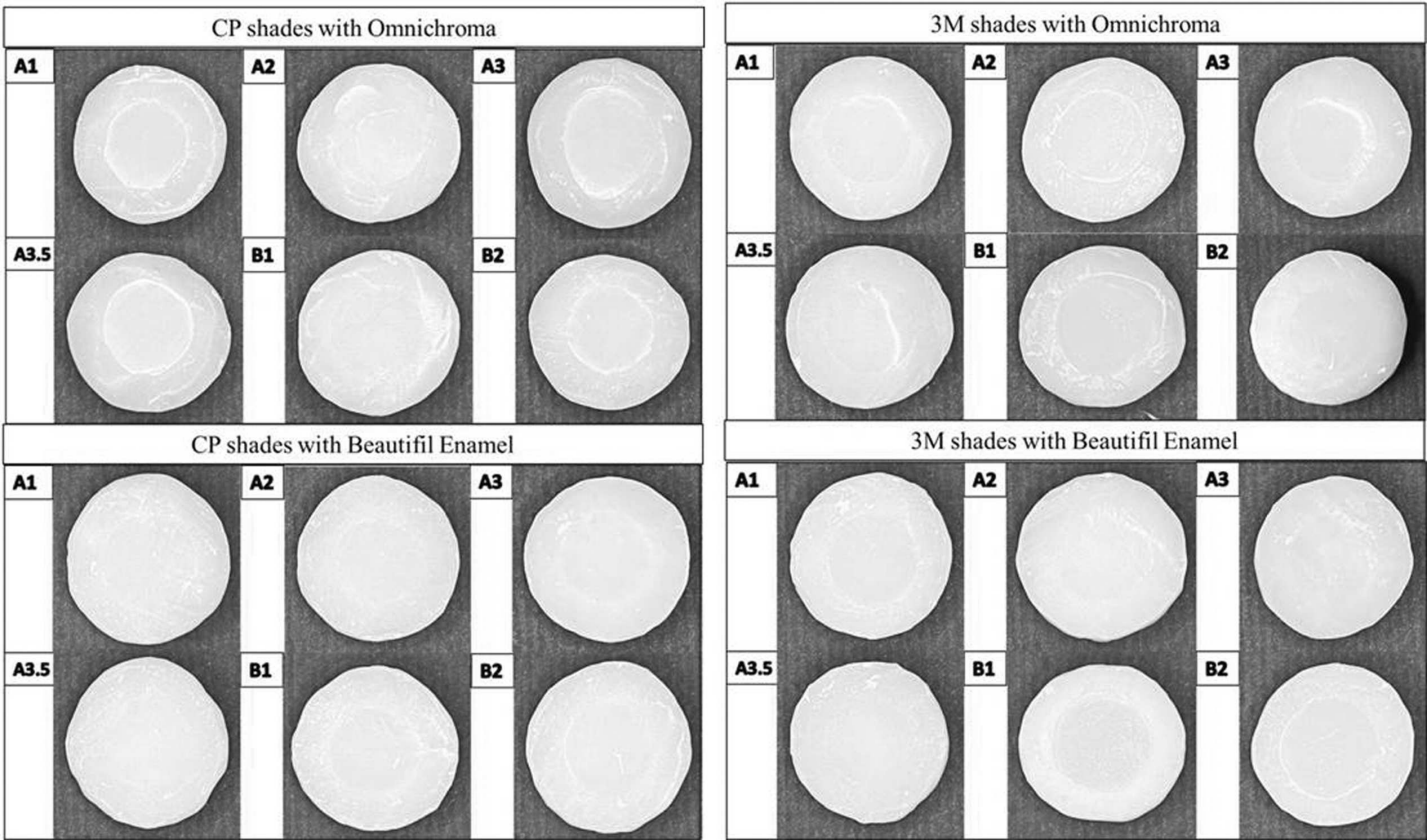
Representative image of CF and 3M shades blending with BE and OM before staining challenges.

The multivariant analysis revealed a statistically significant influence of materials ( *p* < 0.001) and shades ( *p* < 0.001) on the color parameters of the composite. Multiple comparison showed a statistically significant difference in L* ( *p* = 0.001), C* ( *p* = 0.002), and H* ( *p* = 0.001) of CP composite compared with OC and BE. The L* value among the shades of CP was statistically insignificant ( *p* = 0.166) and the OC and BE showed similar pattern when blended with CP. The C* values of A3 and A3.5 shades of CP were significantly different compared with A1 shade ( *p* = 0.013), B1 shade ( *p* = 0.001), and B2 shade ( *p* = 0.01). The C* value of BE blended with A1 and B1 of CP showed a significant difference with that of blended with remaining shades ( *p* < 0.05). The C* value of OC blended with B1 of CP showed a significant difference with that of blended with A3 and A3.5 shades ( *p* < 0.05). The H* values of A3 and A3.5 shades of CP were significantly different compared with A1, A2, B1, and B2 shades ( *p* < 0.05). The H* value of BE showed similar pattern when blended with CP; however, OC did not show any significant difference in H* value when blended with CP. The L*, C*, and H* of each shade of CP, OC, and BE are shown in Fig. 1 . The representative image of each shade of CP with OC and BE is shown in Fig. 2 .

### Blending Effect with 3M

Multiple comparison showed a statistically significant difference in L* ( *p* = 0.002), C* ( *p* = 0.002), and H* ( *p* = 0.001) of 3M compared with OM and BE. The L* values of A3 and A3.5 shades of 3M were significantly different compared with A1, A2, B1, and B2 shades ( *p* < 0.05). The L* value of OC blended with B1 and B2 shades of 3M showed a significant difference ( *p* < 0.05) from that of OC blended with other shades of 3M. The L* value of BE blended with A3.5 shade of 3M showed a significant difference from that of BE blended with other shades of 3M. The C* value of A3.5 shade of 3M was significantly different ( *p* < 0.05) compared with other shades of the same. The C* value of OM blended with B1 shade of 3M showed a significant difference from ( *p* < 0.05) that of OM blended with other shades of 3M. The C* value of BE blended with A1 shade of 3M showed a significant difference ( *p* < 0.05) from that of BE blended with other shades of 3M. The H* values of A3 and A3.5 shades of 3M were significantly different ( *p* < 0.05) compared with other shades of the same. The H* value of OM blended with B1 shade of 3M showed a significant difference from ( *p* < 0.05) that of OM blended with other shades of 3M. The H* value of BE blended with A1 shade of 3M showed a significant difference ( *p* < 0.05) from that of BE blended with other shades of 3M. The L*, C*, and H* of each shade of 3M, OC, and BE are shown in Fig. 1 . The representative image of each shade of 3M with OC and BE is shown in Fig. 2 .

### Staining Challenge

**Fig. 3:**
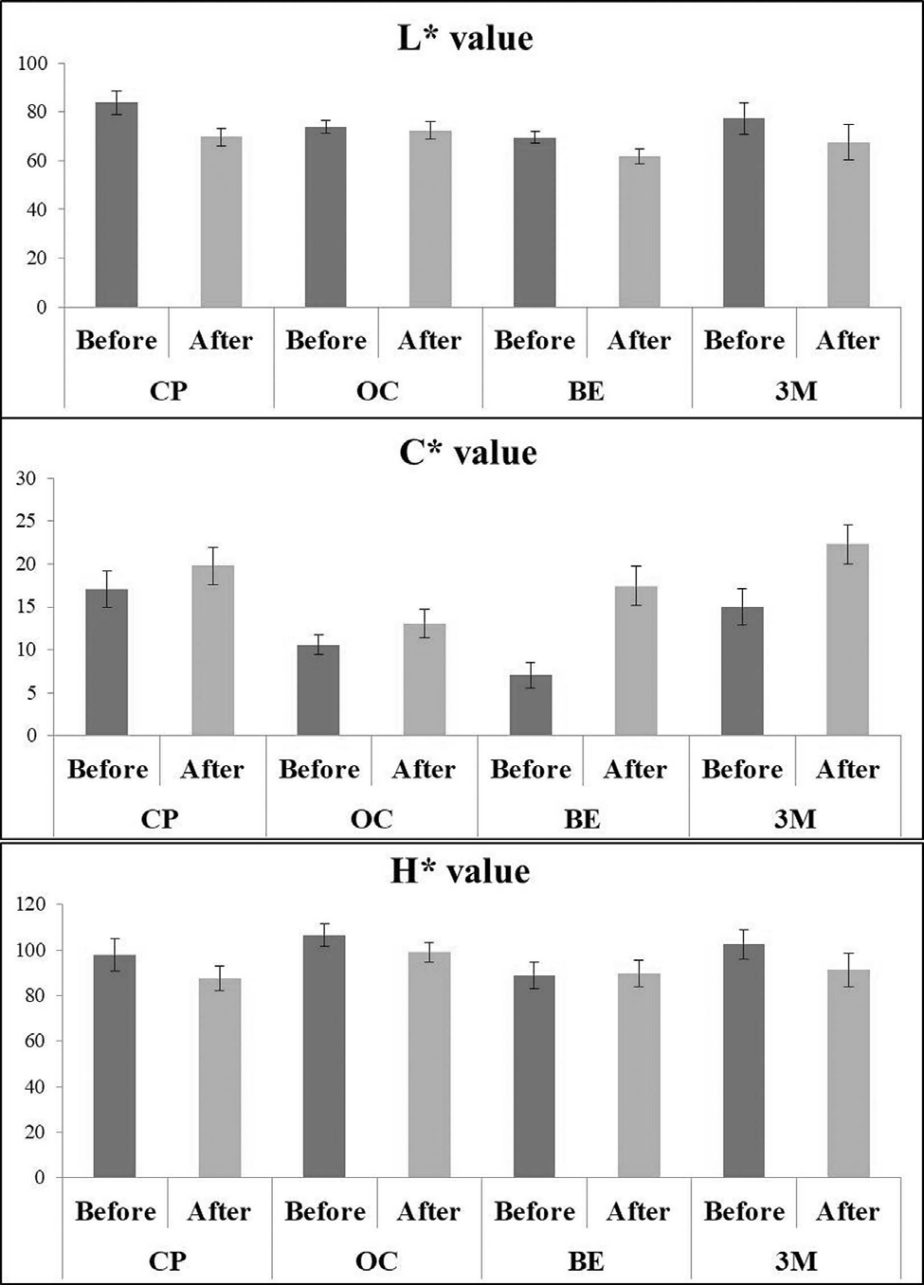
The lightness, chroma, and hue of CF, BE, OM, and 3M before and after staining challenges.

**Fig. 4:**
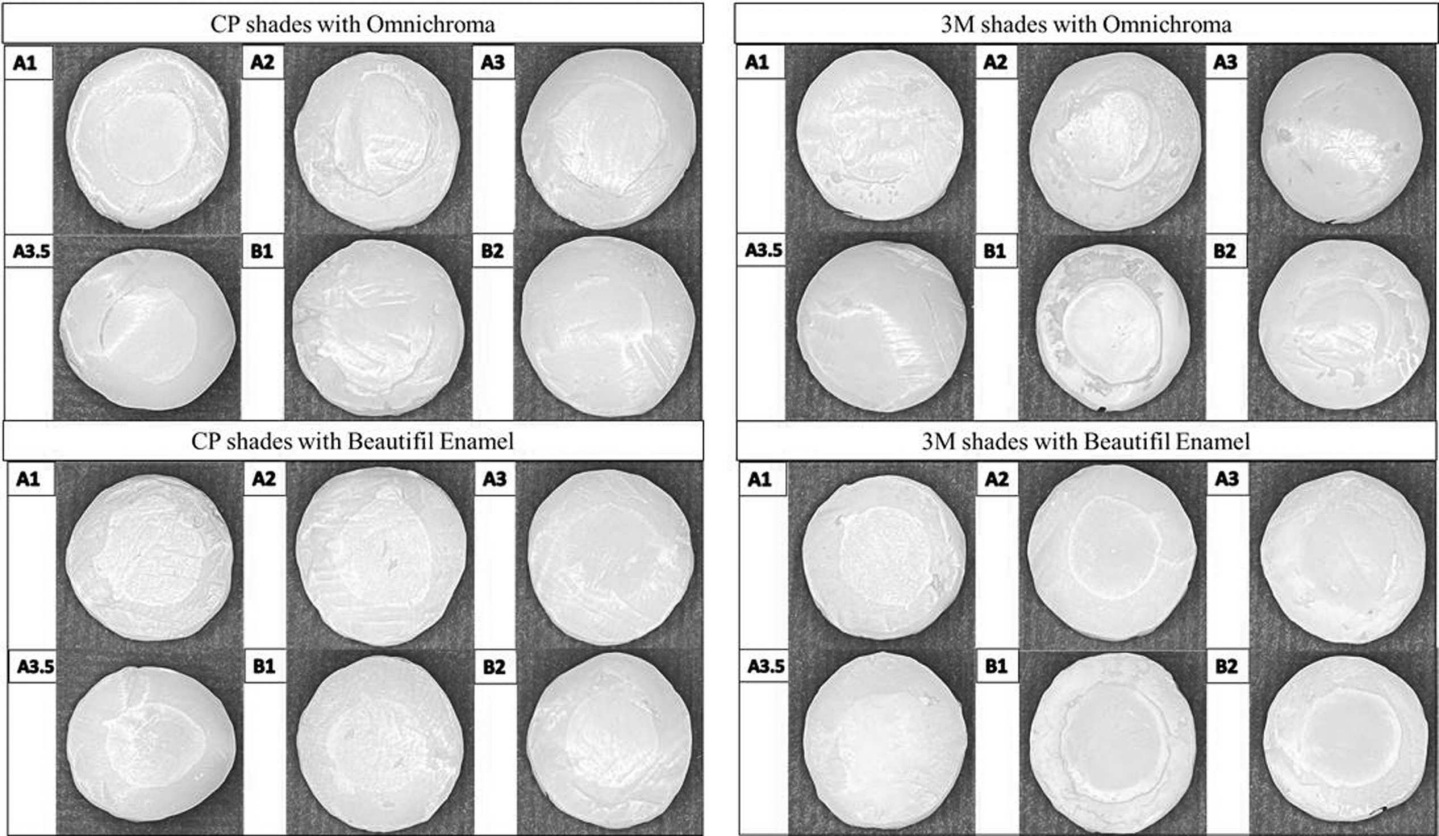
Representative image of CF and 3M shades blending with BE and OM after staining challenges.

The repeated measure ANOVA showed statistically significant ( *p* = 0.001) alteration of color parameters after staining challenge. The mean L* value significantly decreased in CP and BE groups ( *p* < 0.05); however, it was not significantly changed in OC and 3M groups ( *p* > 0.05). The mean C* value significantly increased in BE and 3M groups ( *p* < 0.05); however, the change was not significant in CP and OM groups ( *p* > 0.05). The mean H* value decreased in all groups; however, it did not reach the level of significance ( *p* > 0.05). The means for L*, C*, and H* alterations before and after staining challenge are shown in Fig. 3 . The representative image of each shade of CP and 3M with OC and BE is shown in Fig. 4 .

## Discussion

The results of the current study showed that both OC and BE could blend with only certain shades of CP and 3M. Thus, the first null hypothesis was partially rejected. The blended shades for OC were different from the blended shades for BE, and thus, the second hypothesis was rejected. The color parameters changed after the staining challenge for all groups which requires the rejection of the third null hypothesis.

Choosing the correct shade is a crucial step in direct restorations of teeth or repairing existing restoration using resin-based composites. Simplification and shortening the chairside procedure followed for shade matching are two of the main reasons behind the use of single-shade resin composite materials that are capable of matching with a wide range of classical shades. 18

The translucency and primary color parameters of a resin composite govern the overall esthetic appearance of the restoration. 19 20 The color phenomenon of resin composite is based on the distinction of wavelengths resulting from fundamental optical processes of diffraction, interference, and scattering. 21 According to the manufacturer, OC emits red-to-yellow structural color identical to the color constituents of a natural tooth. As this red-to-yellow structural color is generated, it merges with the reflected light and the color of the surrounding tooth in an additive color mixing process, thus maximizing OC's ability to tone with natural teeth. 22 A previous study by Dunn concluded that OC matched both visually as well as with the instrumented evaluation values of extracted teeth of varying shades (A1–D4). 22 Peyton in 2019 reported that the use of an OC made the whole restorative treatment easier and saved a lot of time by blending the color with natural tooth. 23 The results of the latter two studies are inconsistent with the results of the present study in which we showed that OC blended with only certain shades of CP and 3M.

Pereira Sanchez et al concluded that OC showed substantial discrepancy in matching due to considerable visual rating of single specimens and instrumental color differences. OC is composed of supranano spherical silicon dioxide and zirconium dioxide fillers. The refractive index (RI) of OC monomers switches from 1.47 to 1.52 after polymerization, thus increasing the transparency and blending capability of the material. 18 Iyer et al showed that OC matched better with lighter shades visually. 24 The findings of their study are consistent with the results reported in our study where we found that OC blended with lighter shades such as B1, A2, and B2 of CP and 3M; however, OC showed contrast with darker shade such as A3 and A3.5. The differences or similarities in the results obtained with the aforementioned studies in comparison with the current study are mainly attributed to the used methodologies.

The color appearance of resin composite can be influenced by the color at the cavity floor, and this effect hugely depends on the translucency of the used resin composite. Translucency is an optical phenomenon of an object that allows the light to be absorbed and partially pass through it. Translucent materials prevent clear vision of background objects by dispersing light. 5 25 The RI of an object is related to the direction of the light and altered with a shift of light direction. In the case of highly translucent resin composites, RI of the inorganic fillers is very close to that of the resin matrix. 26 In order to achieve a pleasing blending effect, the RI of matrix and filler need to match; otherwise, the filler may amplify the opacity by prompting refraction at the filler–matrix interface. 27

With respect to providing clinicians with cost-effective alternatives to ceramic veneer, Giomer technology is incorporated within the BE system for a chameleon-like optical characteristics to attain polychromatic restorations that are esthetically and morphologically identical to natural teeth. The result of our study showed that the BE can blend with certain shade of resin composite and the blended shades were different for CP when compared with 3M. The speculated explanation of this dissimilarity is the variations in the fillers and organic matrix of the latter two resin composite systems. 26

Paravina et al reported that smaller size cavities exhibit better blending effect compared with larger cavities. Moreover, the blending effect diminishes in cases where there is large contrast between the restoration and the surroundings. 28 The blending effect of both OC and BE in our study was limited to only certain shades which could be attributed to the use of large size window in the resin composite cylinder. The color stability of resin composite depends on multiple factors including the size and types of fillers, finishing and polishing of composite surface, and filler:matrix ratio. 29 30 31 The results of the staining challenge in our study might have been influenced by the lack of polishing of composite surfaces and the differences in the composition of the used materials which could have resulted in variations in the degree of color alteration.

## Conclusion

The *in vitro* experimental conditions used in this study is considered one of the limitations as it gives rise to only limited answers to more complex problems. Considering the limitations of *in vitro* studies and the sensitivity of the spectrophotometer, it may be concluded that OC and BE blend with only certain shades of conventional resin composite, and therefore, this might call for careful use of single-shade resin composite especially for the repair of an existing composite or tooth structure of darker shades. However, the blending effect of the tested materials might be different in a clinical environment, and further studies must be conducted to evaluate the performance of single-shade resin composite systems clinically in different conditions.

## References

[1] Lavigueur C Zhu X X Recent advances in the development of dental composite resins RSC Adv 2012 2 01 59 63

[2] Aminoroaya A Neisiany R E Khorasani S N A review of dental composites: challenges, chemistry aspects, filler influences, and future insights Compos Part B Eng 2021 216 108852

[3] Lee Y-K Translucency of human teeth and dental restorative materials and its clinical relevance J Biomed Opt 2015 20 04 45002 10.1117/1.JBO.20.4.045002 25875626

[4] Alzraikat H Burrow M F Maghaireh G A Taha N A Nanofilled resin composite properties and clinical performance: a review Oper Dent 2018 43 04 E173 E190 29570020 10.2341/17-208-T

[5] Villarroel M Fahl N De Sousa A M De Oliveira O B Jr Direct esthetic restorations based on translucency and opacity of composite resins J Esthet Restor Dent 2011 23 02 73 87 21477031 10.1111/j.1708-8240.2010.00392.x

[6] Ceci M Viola M Rattalino D Beltrami R Colombo M Poggio C Discoloration of different esthetic restorative materials: a spectrophotometric evaluation Eur J Dent 2017 11 02 149 156 28729784 10.4103/ejd.ejd_313_16 PMC5502556

[7] Kalantari M H Ghoraishian S A Mohaghegh M Evaluation of accuracy of shade selection using two spectrophotometer systems: Vita Easyshade and Degudent Shadepilot Eur J Dent 2017 11 02 196 200 28729792 10.4103/ejd.ejd_195_16 PMC5502564

[8] Yamaguchi S Karaer O Lee C Sakai T Imazato S Color matching ability of resin composites incorporating supra-nano spherical filler producing structural color Dent Mater 2021 37 05 e269 e275 33563472 10.1016/j.dental.2021.01.023

[9] Bakti I Santosa A S Irawan B Damiyanti M Chameleon effect of nano-filled composite resin restorations in artificial acrylic teeth of various shades J Phys: Conf Ser 2018 1073 52011

[10] Abdelraouf R M Habib N A Color-matching and blending-effect of universal shade bulk-fill-resin-composite in resin-composite-models and natural teeth BioMed Res Int 2016 2016 4.183432E6 10.1155/2016/4183432 PMC5097793 27843942

[11] Mourouzis P Koulaouzidou E A Palaghias G Helvatjoglu-Antoniades M Color match of resin composites to intact tooth structure J Appl Biomater Funct Mater 2015 13 03 e259 e265 26108430 10.5301/jabfm.5000228

[12] Lee Y K Lim B S Kim C W Difference in the colour and colour change of dental resin composites by the background J Oral Rehabil 2005 32 03 227 233 15707434 10.1111/j.1365-2842.2004.01402.x

[13] Durand L B Ruiz-López J Perez B G Color, lightness, chroma, hue, and translucency adjustment potential of resin composites using CIEDE2000 color difference formula J Esthet Restor Dent 2021 33 06 836 843 33283966 10.1111/jerd.12689

[14] Eliezer R Devendra C Ravi N Tangutoori T Yesh S Omnichroma: one composite to rule them all Int J Med Sci 2020 7 06 6 8

[15] Lowe R A OMNICHROMA: one composite that covers all shades for an anterior tooth Compend Contin Educ Dent 2019 40(1, suppl 1):8 10 31478684

[16] Auster P Evolution and revolution: groundbreaking changes in composite dentistryDentistry Today 2019;38(02). Available at:https://www.dentistrytoday.com/restorative-134/105

[17] Gingiva and enamel shades for greater individuality Br Dent J 2019 227 04 323 10.1038/s41415-021-3603-x 34686828

[18] Pereira Sanchez N Powers J M Paravina R D Instrumental and visual evaluation of the color adjustment potential of resin composites J Esthet Restor Dent 2019 31 05 465 470 31095870 10.1111/jerd.12488

[19] dos Santos G B Alto R VM Filho H RS da Silva E M Fellows C E Light transmission on dental resin composites Dent Mater 2008 24 05 571 576 17689605 10.1016/j.dental.2007.06.015

[20] Watts D C Cash A J Analysis of optical transmission by 400-500 nm visible light into aesthetic dental biomaterials J Dent 1994 22 02 112 117 8195476 10.1016/0300-5712(94)90014-0

[21] Dumanli A G Savin T Recent advances in the biomimicry of structural colours Chem Soc Rev 2016 45 24 6698 6724 27510041 10.1039/c6cs00129g

[22] Mohamed M A Afutu R Tran D Shade-matching capacity of Omnichroma in anterior restorationsOpen Access J Dent Sci 2020;5(02):

[23] Peyton D J Show Your Work: Restoring Large Class III Cavities with a One-Shade CompositeDentaltown (December 2019) 28–30. Available at:https://www.dentaltown.com/magazine/article/7843/s

[24] Iyer R S Babani V R Yaman P Dennison J Color match using instrumental and visual methods for single, group, and multi-shade composite resins J Esthet Restor Dent 2021 33 02 394 400 32844567 10.1111/jerd.12621

[25] Kolb C Gumpert K Wolter H Sextl G Highly translucent dental resin composites through refractive index adaption using zirconium dioxide nanoparticles and organic functionalization Dent Mater 2020 36 10 1332 1342 32736851 10.1016/j.dental.2020.07.005

[26] Ota M Ando S Endo H Ogura Y Miyazaki M Hosoya Y Influence of refractive index on optical parameters of experimental resin composites Acta Odontol Scand 2012 70 05 362 367 21780980 10.3109/00016357.2011.600724

[27] Arai Y Kurokawa H Takamizawa T Evaluation of structural coloration of experimental flowable resin composites J Esthet Restor Dent 2021 33 02 284 293 33098228 10.1111/jerd.12674

[28] Paravina R D Westland S Imai F H Kimura M Powers J M Evaluation of blending effect of composites related to restoration size Dent Mater 2006 22 04 299 307 16085303 10.1016/j.dental.2005.04.022

[29] Barakat O Abbas M Effect of different finishing and polishing systems on surface roughness and color changes of resin composites: an *in vitro* study Egypt Dent J 2019 65 01 657 666

[30] Yildiz E Sirin Karaarslan E Simsek M Ozsevik A S Usumez A Color stability and surface roughness of polished anterior restorative materials Dent Mater J 2015 34 05 629 639 25925685 10.4012/dmj.2014-344

[31] Aydın N Topçu F T Karaoğlanoğlu S Oktay E A Erdemir U Effect of finishing and polishing systems on the surface roughness and color change of composite resins J Clin Exp Dent 2021 13 05 e446 e454 33981391 10.4317/jced.58011 PMC8106933

